# A variant in *KCNQ1* gene predicts metabolic syndrome among northern urban Han Chinese women

**DOI:** 10.1186/s12881-018-0652-3

**Published:** 2018-08-29

**Authors:** Yafei Liu, Chunxia Wang, Yafei Chen, Zhongshang Yuan, Tao Yu, Wenchao Zhang, Fang Tang, Jianhua Gu, Qinqin Xu, Xiaotong Chi, Lijie Ding, Fuzhong Xue, Chengqi Zhang

**Affiliations:** 1 Division of Biostatistics, School of Public Health, Shandong University, 44 Wenhua Xilu, Jinan, 250010 Shandong China; 2 Shandong Provincial Qianfoshan Hospital, Shandong University, 16766 Jingshi Rd, Jinan, 250014 China; 3 Jinan Kingmed Center for Clinical Laboratory Co, Ltd., 554 Zhengfeng Rd, Jinan, 250010 Shandong China; 4 Linyi Centre for Adverse Drug Reaction Monitoring, Linyi, 276000 Shandong China; 5 Department of Imaging and Nuclear Medicine, Taishan Medical University, 619 Changcheng Rd, Tai’an, 271016 Shandong China

**Keywords:** Metabolic syndrome, *KCNQ1*, Single nucleotide polymorphism (SNP), Cohort study

## Abstract

**Background:**

Previous studies have reported that the potassium voltage-gated channel subfamily Q member 1 (*KCNQ1*) gene is associated with diabetes in both European and Asian population. This study aims to find a predictable single nucleotide polymorphism (SNP) to predict the risk of metabolic syndrome (MetS) through investigating the association of SNP in *KCNQ1* gene with MetS in Han Chinese women of northern urban area.

**Methods:**

Six SNPs were selected and genotyped in 1381 unrelated women aged 21 and above, who have had physical check-up in Shandong Provincial Qianfoshan Hospital. Cox proportional model was conducted to access the association between SNPs and MetS.

**Results:**

Sixty one women developed MetS between 2010 and 2015 during the 3055 person-year of follow-up. The cumulative incidence density was 19.964/1000 person-year. The SNP rs163182 was associated with MetS both in the additive genetic model (*RR* = 1.658, *95% CI*: 1.144–2.402) and in the recessive genetic model (*RR* = 2.461, *95% CI*: 1.347–4.496). It remained significant after adjustment. This relationship was also observed in MetS components (BMI and SBP).

**Conclusion:**

A novel association between rs163182 and MetS was found in this study, which can predict the occurrence of MetS among northern urban Han Chinese women. More investigations are needed to be done to assess the possible pathway in which *KCNQ1* gene affects MetS.

**Electronic supplementary material:**

The online version of this article (10.1186/s12881-018-0652-3) contains supplementary material, which is available to authorized users.

## Background

Metabolic Syndrome (MetS) is a complex disorder that is characterized by obesity, hyperglycemia, dyslipidemia, hypertension and insulin resistance [1]. It can increase the risk of type 2 diabetes and cardiovascular disease (CVD) [2]. In recent years, the prevalence of MetS is high (26.8% in females) in urban areas of China, and sex heterogeneity has been found in the relationships between risk factors and MetS [3]. Much research of Genome-Wide Association Studies (GWAS) and candidate gene studies have been conducted in Indian [4], Finnish [5], Chinese [6–9], Taiwanese and Caucasian youth [10]. However, these mechanisms were not consistent among the previous studies, and most of the studies were based on cross-sectional studies. Thus, this study aimed to investigate whether the selected SNPs would be associated with MetS in Chinese women based on a cohort study.

*KCNQ1* is a gene that provides instructions for making potassium channels. The gene is expressed in a wild variety of tissues such as cardiac muscle, inner ear, kidney, lung, stomach, and intestine. Cardiac long QT syndrome and congenital deafness are associated with *KCNQ1* gene. However, *KCNQ1* is also expressed in pancreas, and it could influence the insulin secretion [11]. SNPs in *KCNQ1* are significantly associated with lower HOMA-B values [12]. Most studies indicated that the *KCNQ1* gene was a diabetes susceptibility gene in different ancestors [13, 14]. Rs231359, rs2237895, rs2237897, rs2237892, and rs231361 polymorphisms were confirmed among Chinese [12, 15, 16]. Type 2 diabetes was the major consequence of MetS [17], and MetS might be an important risk factor for type 2 diabetes [18]. Meanwhile, insulin resistance is a central feature of MetS and the function of the polymorphisms in *KCNQ1* gene for MetS has not been investigated. Previous research had confirmed that both genetic and environmental factors contribute to the pathogenesis of MetS [1, 19]. Some studies only involved few factors such as smoking or drinking.

In this study, we established a cohort study with 1381 females based on the routine health check-up systems in the urban Han Chinese. We aimed to investigate the association among the selected SNPs in the *KCNQ1* gene with MetS in Chinese women after adjusting for potential confounding variables, as well as to provide a genetic basis to establish a prediction model of MetS for the personalized health management for women. Meanwhile, we also explored the relationship between SNP and MetS components.

## Methods

### Study subjects

This was a cohort study based on the Center for Health Management of Shandong Provincial Qianfoshan Hospital. The health examination database contained persons in Jinan, representing the middle to upper class population of Shandong Province [20, 21]. The participants had two or more records from 2010 to 2015. We selected by a simple random sampling from those who was free of MetS or cardiovascular disease on the first physical examination, and collected their blood samples from April to September 2016. A standardized questionnaire was used to investigate the environmental and dietary risk factors (Additional file 1). Their fasting blood samples were collected and stored in an ultra-low temperature freezer. A total of 1381 women (aged 21 to 81) were included. All individuals were Han Chinese and they were genetically unrelated to each other.

The study was approved by the Ethics Committee of School of Public Health, Shandong University (the number: 20120315). Written informed consent was obtained from all of the participants before the study.

### Measurements

The questionnaire, anthropometric and fasting laboratory assay were conducted in each participant. Although there was a general questionnaire in the original cohort [22], the details of lifestyle factors in women were restricted. A detailed questionnaire was applied to these women, including basic demographic information (age, education and occupation), physiological condition (menarche, dysmenorrhea, menopause and reproductive system disease), marriage and pregnancy situation (marriage age, the number of births and abortions), lifestyles (taste preference, sleep, housework, smoking, secondhand smoking and drinking) and family medical history (diabetes, hypertension, obesity, hyperlipidemia and CVDs). Food Frequency Questionnaire (FFQ) was also conducted by asking dietary habits within one month. Some details of the questionnaire are shown in Additional file 2: Table S1.

The anthropometric measurements included weight, height and blood pressure. Weight and height were measured by standardized procedures. Body mass index (BMI) was calculated in accordance with weight/height^2^ (kg/m^2^). Blood pressure was measured on the right arm by an automated sphygmomanometer after 5-min rest. The biomarkers related to MetS, including systolic blood pressure (SBP), diastolic blood pressure (DBP), fasting plasma glucose (FPG), total cholesterol (TC), triglyceride (TG), low-density lipoprotein-cholesterol (LDL-C) and high-density lipoprotein cholesterol (HDL-C), were measured by the standard clinical and laboratory protocol in the Center for Health Management of Shandong Provincial Qianfoshan Hospital, which was described previously [23, 24].

### Diagnosis of MetS

Considering the practical conditions of physical examination management in Shandong Province and the physiological characteristics of our study subjects, the diagnostic criterion adopted in this study is the one that is recommended by Diabetes Society of the Chinese Medical Association (CDS) [25]. MetS was defined as three or more of the following disorders: (1) overweight or obesity (BMI ≥ 25.0 kg/M^2^); (2) hypertension (SBP ≥ 140 mmHg, DBP ≥ 90 mmHg or diagnosed before); (3) hyperglycemia (FPG ≥ 6.1 mmol/L or 2 h post-meal glucose ≥7.8 mmol/L, or diagnosed before); and (4) dyslipidemia (TG ≥ 1.7 mmol/L, or HDL < 1.0 mmol/L in female).

### SNP selection, genotyping and quality control analysis

We selected six SNPs (rs2237892, rs231361, rs2237895, rs231359, rs2237897 and rs163182) from the *KCNQ1* gene, which were reported to be associated with diabetes [15, 18, 26] . The minor allele frequency (MAF) of the above six SNPs was greater than 0.05 in the Chinese Han population from the NCBI dbSNPs database (http://www.ncbi.nlm.nih.gov/). DNA was extracted from venous blood samples, which were collected in the morning after 8 h of fasting, and then stored in an ultra-low temperature freezer. Genomic DNA extraction and genotyping were accomplished by the company, BioMiao Biological Technology (Beijing) Co., Ltd.

### Statistical analyses

Taking missing values of clinical covariates into account, multiple imputations were performed using Amelia II by R3.3.2. All imputation variables had less than 10% missing observations before imputation.

The Hardy-Weinberg Equilibrium (HWE) of six SNPs was performed utilizing the Chi-squared test by R3.3.2. The linkage disequilibrium (LD) was performed by Haploview4.2. Markers of SNP were not included in the analysis, in the conditions that the call rate was less than 95%, and the *P* value from a test of HWE was less than 0.05.

Continuous variables were described with mean (standard deviation) and categorical variables were summarized as percentages. Differences in the baseline between MetS and non-MetS during the follow-up were compared using Student’s *t-*test for continuous variables, and Chi-squared test for categorical variables. The Cox proportional hazards model was utilized to discover the association of SNPs in the additive model, dominant model and recessive model after adjusting for potential environmental confounding. The differences between SNPs and metabolic syndrome components were analyzed by the covariance analysis. Aiming to investigate whether SNPs could contribute to the efficiency of the prediction model, we compared the area under receiver operator characteristic curve (AUC) between with and without SNP. A two-tailed *P* value of less than 0.05 was regarded as statistically significant. These statistical analyses were performed using SAS version 9.4.

## Results

### Basic characteristics

This cohort (*n* = 1381) had a mean age of 39.5 at baseline. During the 3055 person-year of follow-up, 61 women developed MetS between 2010 and 2015. The cumulative incidence density was 19.964/1000 person-year. The baseline characteristics of metabolic syndrome components for participants are shown in Table 1. Cases had significantly higher BMI, SBP, DBP, FPG, TG, TC and LDL-C, but lower HDL-C than controls.

**Table 1:** Baseline clinical characteristics of the women according to the MetS status

	MetS (*n* = 61)	Non-MetS (*n* = 1320)	*T* test	*P*
Age (year)	53.984 ± 12.027	38.812 ± 11.621	−9.950	< 0.001
BMI (kg/m2)	26.588 ± 3.136	22.079 ± 2.923	−11.740	< 0.001
SBP (mmHg)	134.180 ± 15.898	118.698 ± 14.650	−5.690	< 0.001
DBP (mmHg)	79.672 ± 9.916	72.471 ± 9.644	−8.040	< 0.001
FPG (mmol/L)	5.483 ± 0.811	4.997 ± 0.501	−4.640	< 0.001
TG (mmol/L)	1.525 ± 0.852	0.927 ± 0.498	−5.440	< 0.001
TC (mmol/L)	5.149 ± 0.803	4.634 ± 0.846	−4.660	< 0.001
LDL-C (mmol/L)	3.131 ± 0.657	2.633 ± 0.670	−5.680	< 0.001
HDL-C(mmol/L)	1.430 ± 0.292	1.600 ± 0.294	4.430	< 0.001

*BMI* indicted body mass index, *SBP* systolic blood pressure, *DBP* diastolic blood pressure, *FPG* fasting plasma glucose *TG* triglycerides, *TC* total cholesterol, *LDL-C* low-density lipoprotein-cholesterol, *HDL-C*, high-density lipoprotein-cholesterol

In this study, women rarely smoked or drank. Besides, secondhand smoking showed no statistical significance with MetS. Additional file 2: Table S1 shows the frequency and percentages of environmental variables of the study. Significant differences in marital status, education, sleep, housework time, trip mode, menopausal status, pregnancy information (the number of births and abortions), reproductive system disease, taste preference, intake of fruit and meat between cases and controls were found by simple Cox proportional model (Additional file 2: Table S2).

### SNPs in KCNQ1 gene and MetS

Rs231361 was not in genotype quality or Hardy-Weinberg equilibrium (*P* < 0.05) (Additional file 2: Table S3). Hence, it was excluded. The genotype and allele distribution of the five SNPs are shown in Table 2. Only rs163182 showed a difference between MetS and non-MetS both in genotypes (*P* = 0.016) and in allele distribution (*P* = 0.010). We carried out LD mapping in Additional file 3: Figure S1. Rs231359 was isolated compared with the other SNPs (*r*^2^ = 0). The *r*^2^ of each pairwise LD among rs2237892, rs163182, rs2237895 and rs2237897 was modest (0.10 < r^2^ < 0.69).

**Table 2:** Distribution of genotype and allele of SNPs in the *KCNQ1* gene

SNP	Genotype	number of Genotype		*P*(G)^a^	Major/minor allele	number of allele		*P* (A)^b^
	0/1/2	MetS	Non-MetS			MetS	Non-MetS	
rs163182	GG/CG/CC	18/26/14	550/593/156	0.016	G/C	62/54	1693/905	0.010
rs231359	AA/CA/CC	40/18/2	814/445/50	0.782	A/C	98/22	2073/545	0.511
rs2237895	AA/CA/CC	23/32/6	609/570/130	0.361	A/C	78/44	1788/830	0.312
rs2237897	CC/CT/TT	23/29/9	561/592/159	0.685	C/T	75/47	1714/910	0.384
rs2237892	CC/CT/TT	25/32/4	619/564/130	0.308	C/T	82/40	1802/824	0.743

^a^The *P* value of Chi-square test for the genotype between MetS and non-MetS

^b^*P* value of Chi-square test for the allele of SNPs

Table 3 shows the association analyses of the five SNPs with MetS by Cox proportional model. Single SNP analysis revealed that the rs163182 was associated with MetS in the additive genetic model (relative risk (*RR*) =1.658, *95% CI*: 1.144–2.402) and the recessive genetic model (*RR* = 2.461, *95% CI*: 1.347–4.496) (Table 3). Other SNPs suggested no association with MetS. After adjusting for age, the results showed that with the increase of the number of C alleles in rs163182 genotype, *RR* was 1.531 (*95%CI*: 1.064–2.204), and *RR* of CG + CC genotype vs GG was 2.086 (*95%CI*: 1.14–3.816). After adjusting for possible confounding factors (age, marital status, education, sleep, housework, trip mode, menopausal status, the number of births, the number of abortions, reproductive system disease, intake of fruit and fresh meat), the SNP remained significantly associated with MetS in the additive genetic model (*RR* = 1.776, *95% CI*: 1.166–2.704) and the recessive genetic model (*RR* = 2.976, *95% CI*: 1.488–5.951) (Table 3). Other four SNPs in the *KCNQ1* gene had no statistical significance in this study (*P* > 0.05).

**Table 3:** The association analyses of the five SNPs with MetS with Cox proportional model

SNP	*RR* (*95% CI*)	*P*	Adjusted *R*^a^ (*95% CI*)	Adjusted *P*	Adjusted *RR*^b^ (*95% CI*)	Adjusted *P*
rs163182						
GG/CG/CC^c^	1.658 (1.144–2.402)	0.008	1.531 (1.064–2.204)	0.022	1.776 (1.166–2.704)	0.007
CG + CC^d^	1.603 (0.919–2.797)	0.096	1.538 (0.881–2.685)	0.130	1.624 (0.878–3.003)	0.122
CC^e^	2.461 (1.347–4.496)	0.003	2.086 (1.14–3.816)	0.017	2.976 (1.488–5.951)	0.002
rs231359						
AA/CA/CC^c^	0.881 (0.553–1.406)	0.596	0.951 (0.596–1.516)	0.832	0.914 (0.551–1.515)	0.727
CA + CC^d^	0.862 (0.504–1.474)	0.586	0.94 (0.549–1.611)	0.823	0.959 (0.538–1.71)	0.887
CC^e^	0.874 (0.213–3.578)	0.851	0.959 (0.234–3.932)	0.954	0.503 (0.068–3.739)	0.502
rs2237895						
AA/CA/CC^c^	1.216 (0.838–1.763)	0.304	1.138 (0.777–1.665)	0.507	1.273 (0.836–1.939)	0.260
CA + CC^d^	1.428 (0.851–2.396)	0.178	1.266 (0.753–2.130)	0.374	1.475 (0.836–2.604)	0.180
CC^e^	0.999 (0.430–2.321)	0.998	0.986 (0.424–2.293)	0.974	1.082 (0.417–2.805)	0.871
rs2237897						
CC/CT/TT^c^	1.188 (0.828–1.705)	0.349	1.187 (0.835–1.687)	0.341	1.296 (0.876–1.915)	0.194
CT + TT^d^	1.267 (0.755–2.126)	0.371	1.341 (0.798–2.253)	0.267	1.383 (0.788–2.43)	0.259
TT ^e^	1.235 (0.608–2.506)	0.559	1.123 (0.553–2.280)	0.747	1.46 (0.687–3.101)	0.325
rs2237892						
CC/CT/TT^c^	1.062 (0.728–1.550)	0.754	1.074 (0.739–1.563)	0.707	1.201 (0.792–1.82)	0.389
CT + TT^d^	1.277 (0.766–2.127)	0.348	1.319 (0.791–2.198)	0.289	1.435 (0.821–2.506)	0.205
TT^e^	0.636 (0.231–1.754)	0.382	0.619 (0.225–1.708)	0.355	0.849 (0.295–2.439)	0.761

^a^Adjusted for age

^b^Adjusted for age, marital status, education, sleep, housework, trip mode, menopausal status, the number of births, the number of abortions, reproductive system disease, intake of fruit and fresh meat

^c^Additive model

^d^Dominant model

^e^Recessive model

Aiming to test and verify the effect of rs136182 on MetS prediction, we compared the performance on two prediction models of MetS using age, menopausal status, the number of births and the intake of fruit, with and without rs163182. The AUC for the prediction model with and without rs163182 was 0.743 (*95% CI*, 0.719–1.766) and 0.756 (*95% CI*, 0.732–0.779) respectively. To a certain extent, the efficiency was improved.

### SNP rs163182 and metabolic syndrome components

The association between rs163182 and metabolic components was also explored (Table 4). The difference between SBP and the genotypes (GG, CG and CC) was analyzed under the covariance analysis after adjusting for age (*P* = 0.043). The differences of other biomarkers DBP, FPG, TG and HDL-C were not detected between each three genotypes. The differences between rs163182 genotype and BMI were conducted by non-parameters Wilcoxon symbols test, which suggested rs163182 was associated with BMI (*P* = 0.032).

**Table 4:** Comparison of metabolic syndrome components between different groups of rs163182 genotypes

	rs163182			*P* ^a^	*P* ^b^
	GG (*n* = 568)	CG (*n* = 619)	CC (*n* = 170)		
SBP	119.083 ± 14.853	118.877 ± 14.580	122.671 ± 16.859	0.043	0.138
DBP	72.541 ± 9.690	72.673 ± 9.654	74.073 ± 10.500	0.378	0.414
FPG	5.008 ± 0.531	5.003 ± 0.494	5.090 ± 0.602	0.323	0.508
TG	0.927 ± 0.495	0.951 ± 0.526	0.999 ± 0.493	0.433	0.854
HDL-C	1.600 ± 0.297	1.596 ± 0.299	1.564 ± 0.281	0.341	0.753
BMI	22.135 ± 2.871	22.237 ± 3.104	22.899 ± 3.502	0.032^c^	0.020

*BMI* indicted body mass index, *SBP* systolic blood pressure, *DBP* diastolic blood pressure, *FPG* fasting plasma glucose, *TG* triglycerides, *TC* total cholesterol, *LDL-C* low-density lipoprotein-cholesterol, *HDL-C* high-density lipoprotein-cholesterol

^a^The *P* value of covariance analysis after adjusting for age

^b^The *P* value of the homogeneity test

^c^The *P* value of non-parameters Wilcoxon symbols test for BMI

## Discussion

In this study, we discovered a novel association between *KCNQ1*-rs163182 and MetS, which suggests that rs163182 is an independent predictor for MetS. Meanwhile, rs163182 was also associated with MetS components (BMI and SBP) in Han Chinese women of northern urban area. Rs163182 may play a role in the biological metabolism.

In the current study, the etiology of MetS refers to environmental confounding factors, genetic susceptibility, as well as their interactions [27, 28]. Identifying genes had the following functions: dramatically improve understanding of the mechanisms of MetS [29], identify people at high risk, and prevent the development of diabetes and CVD. The relationships among SNPs, environmental factors and MetS had received considerable attention. In our study, we reviewed a large amount of literature about SNPs for MetS and its components (obesity, hyperglycemia, dyslipidemia, hypertension and insulin resistance). According to the preliminary study, rs163182, which carried C-allele in the *KCNQ1* gene, was more likely to develop MetS. In addition, it remained statistically significant after adjusting for potential risk factors. That indicates rs163182 in the *KCNQ1* gene is a novel independent predictor for MetS.

It has been confirmed that the *KCNQ1* gene was associated with diabetes in population of both Asian and European descent [13, 14, 30]. Meanwhile, there were few studies about rs163182 [15, 31]. In a genome-wide association study for type 2 diabetes in Han Chinese [15], it validated the association between rs163182 and diabetes (*OR* = 1.28), which was conducted in southern China. However, there was a heterogeneity compared with our study. In our study, the *KCNQ1* rs2237892, rs231361, rs2237895, rs231359, rs2237897, rs163182 and many environmental factors were surveyed. Although we did not find positive results of FPG, we discovered a novel association between rs163182 and MetS. In the study by Chen et al. [32], the *KCNQ1* gene was associated with lipid parameters, TG, HDL-C, and apo A1 in a middle-aged Chinese Han population. A possibility has been mentioned that *KCNQ1* may be a molecule affecting insulin sensitivity [33, 34]. The gene of *KCNQ1* not only plays an important role in blood glucose metabolism but also regulates other metabolic substances. The result that rs163182-C would increase the risk of MetS’ occurrence was understood. On the other hand, referring to the causal inference [35], MetS was a risk factor for diabetes and the *KCNQ1* gene was associated with diabetes. Thus, the *KCNQ1* gene may influence the occurrence of MetS.

We also investigated the role of environmental variables to MetS. Widow or divorce, more housework, peri-menopause or menopause, multiple pregnancies (including birth and abortion), and reproductive system disease would increase the risk of MetS (*RR* > 1.0), while highly-educated people, normal diet (means the taste preference is reasonable), and more fruit or meat would reduce the onset of MetS. That suggested the lifestyle or the information of fertility may influence the MetS, which was consistent with previous studies [36, 37]. We also explored the interactions between rs163182 and these factors, of which the results were negative. That indicates the interactions between rs163182 and environmental factors to MetS are a minor effect.

Aiming to evaluate the prediction effect of rs163182, we calculated AUC with and without rs163182. The result reveals the *KCNQ1* gene may provide a new method for modeling a risk prediction for MetS, which can use rs163182 to achieve the personalized health management in Han Chinese women of northern urban area.

The association between rs163182 and MetS components was also researched. The statistical significance was found among BMI and SBP in rs163182 genotype. The study of Sinha et al. [38] used *KCNQ1* to exhibit both differential methylation and differential gene expression by comparing adipocytes between obese and never-obese women. The *KCNQ1* plays roles in cardiac tissue. Mutations in this gene may impair the function of heart. However, the specific mechanisms need to be further validated.

Considering the survival data in this study, Cox proportional model was applied. To our knowledge, few investigations were conducted by cohort study using Cox proportional model [39] adjusting for other potential variables.

The study also has several limitations. Firstly, the participants comprised only women who came to the Center for Health Management of Shandong Provincial Qianfoshan Hospital, and might not represent the general population. In some way, it could restrict female to avoid gender confounding. Secondly, the time of follow-up in this study was not long enough, that result in the limited number of cases. So this study will be followed up continuously in the future. Thirdly, because of the large population in the Center for Health Management, the subjects were selected simply and randomly based on the database that may be a limitation in some way. In the follow-up study, a more sophisticated strategy could be employed to verify the result, such as a nest case-control study.

## Conclusions

It was the first time to discover that rs163182 in *KCNQ1* gene would raise the risk of MetS and elevate the level of BMI and SBP. It partly explains the mechanism of MetS and may provide a new comprehension of molecular mechanism. Of course, further research is needed to evaluate the genetic marker in different populations.

## Additional files

Additional file 1: Questionnaire of health behavior of Han Chinese women in Shandong Province. (DOCX 20 kb)

Additional file 2: **Table S1.** Environmental characteristics of the subjects in Chinese Women in baseline. **Table S2.** The results of simple cox proportional model for environmental confounding factors and MetS. **Table S3.** Genotype quality and Hardy-Weinberg equilibrium (HWE) of six SNPs in *KCNQ1* gene. (XLSX 18 kb)

Additional file 3: **Figure S1.** the map (*r*^*2*^) of linkage disequilibrium of *KCNQ1* gene SNPs rs231359, rs2237892, rs163182, rs2237895 and rs2237897. (DOCX 19 kb)

## Abbreviations

AUC: Area under receiver operator characteristic curve
BMI: Body mass index
CI: Confidence interval
CVD: Cardiovascular disease
DBP: Diastolic blood pressure
FPG: Fasting plasma glucose
GWAS: Genome Wide Association Study
HDL-C: High-density lipoprotein-cholesterol
HWE: Hardy-Weinberg Equilibrium
KCNQ1: potassium voltage-gated channel subfamily Q member 1
LDL-C: low-density lipoprotein-cholesterol
MAF: Minor allele frequency
MetS: Metabolic syndrome
RR: Relative risk
SBP: Systolic blood pressure
SNP: Single nucleotide polymorphisms
TC: Total cholesterol
TG: Triglycerides
