# Supplementary material for: A variant in KCNQ1 gene predicts metabolic syndrome among northern urban Han Chinese women
Source: BMC Med Genet. 2018 Aug 29;19:153. doi: 10.1186/s12881-018-0652-3 (PMC6114251; doi:10.1186/s12881-018-0652-3)
Supplement: Supplementary file 1 — Questionnaire of health behavior of Han Chinese women in Shandong Province. (DOCX 20 kb) [file 12881_2018_652_MOESM1_ESM.docx]

QUESTIONNAIRE OF HEALTH BEHAVIOUR OF HAN CHINESE WOMEN IN SHANDONG PROVINCE

A. General Information

1. Date of birth: __________

2. Height ______cm Weight____ kg

3. Marital status: (1) Single (2) Married (3) Divorced or Widowed

4. Nation: (1) Han (2) Minorities: _______

5. Occupation: (1) Organizational staff (2) Corporate company leaders or staff (3) Teachers

(4) Medical staff (5) Scientific research

(6) merchants (7) Workers (8) Peasants

(9) unemployed (10) Retired (11) Students (12) Others

6. Education level : (1) Elementary/primary (2) high school/secondary school

(3) undergraduate/junior college (4) master degree or above.

**B Lifestyle and behavior**

*Smoking status*

1. Do you smoke cigarettes? (Smoking more than one a day and lasting 6 months consecutively):

(1) Never (2) Yes, average __ cigarettes/day, lasts for ___ years

(3) used to, smoking cessation ____ years

2. Passive smoking (smokers do not answer this question)

(1) Almost no (2) Less than 1 day / week (3) About 1-2 days / week

(4) About 3-4 days / week (5) Almost daily

*Drinking*

3. Do you drink alcohol:

(1) No

(2) used to, you quit drinking already ______ years

(3) drinking, drinking at______ years old, type: _______, frequency: ______ times / month

4. Do you drink tea:

(1) No (2) 1-2 times / week (3) 3-5 times / week (4)> 5 times / week

5. Beginning at ______ years old

6. What kind of tea do you usually drink? __________________ (Please write the name of the tea, such as black tea, green tea, etc.)

*Activity*

7. Whether your daily life is regular: (1) No (2) Yes

8. How much sleep do you need ____ hours,

And sleeping quality: (1) good (2) better (3) general (4) not so good (5) not good

9 types of work: (the daily working time is divided into mental and physical labor)

(1) mental work: working __ hours a day

(2) manual labor: a. Mild b. Moderate c. Severe, working ______ hours per day

10. The average daily housework time: ________ hours

11. Daily sit-in time: ________ hours (including at work or home watching computers, television time)

12. Mode of travel: (1) Walking (2) Bike (3) Motorbike / Electric car (4) Buses (5) By car

13. Regular exercise: exercise mode: _______

(1) never go to exercise (2) 1-2 times / week (3) 3-5 times / week (4)> 5 times / week

**C Diseases of personal history and family history**

*Personal history*

1.Hypertension, the time of diagnosis: __________ ; whether to use drugs: (1) no (2) yes;

2. diabetes, the time of diagnosis: ______________; whether to use drugs: (1) no (2) yes;

3. Hyperlipidemia, the time of diagnosis: ________; whether to use drugs: (1) no (2) yes;

4. Other Diseases _______, the time of diagnosis: _____________; Drugs Used: (1) No (2) Yes;

*Family history (please tick √ in the appropriate box)*

| Disease | Father | Mother | Brothers /sisters | Your children |
| --- | --- | --- | --- | --- |
| 1. Hypertension |  |  |  |  |
| 2. Hyperlipidemia |  |  |  |  |
| 3. Diabetes |  |  |  |  |
| 4. Obesity |  |  |  |  |
| 5.Coronary heart disease |  |  |  |  |
| 6. Stroke |  |  |  |  |
| 7.Kidney disease |  |  |  |  |
| 8.Other disease_________ |  |  |  |  |

**D gynecological conditions**

*menstrual situation*

1. Menarche ____ years old

2. Whether menstruation is regularly:

(1) regular: a. Menstrual cycle: ___ days b. menstrual period: ___ days (2) irregular

3. The number of sanitary napkins one day during menstrual period?

(1) <3 times (2) 3-5 times (3)> 5 times

4. Dysmenorrhea: (1) No (2) Occasionally (3) Often

5. Menopause: (1) No (2) Yes: menopause at ____ year-old

*reproductive history*

6. Married (first marriage) at _______ years old

7. Have you ever been pregnant (including miscarriage)?

 (1) No (Go to Question 14)

(2) Yes, a total of _____ times of pregnancy, of which: cesarean section ____ times, natural births ____ times, abortion ____ times

8. Your first birth at___ years old (excluding abortion)

9. The lastly birth that you born at____years old?

10. Have you ever had gestational diabetes? (1) No (2) Yes

11. Have you ever had pregnancy-induced hypertension? (1) No (2) Yes

12. Breast-feeding: (1) breast-feeding, a total of __months (2) artificial feeding (milk powder)

(3) mixed feeding (breast milk + milk powder), of which breastfeeding a total of __ months

13. Contraception: (1) No (2) Yes: a. Contraceptives b. IUD c. Condoms

*Gynecological diseases*

14. Breast disease: (1) No (2) Yes, _________

15. Reproductive Diseases: (1) No (2) Yes, ________

**E diet situations**

Note: Select frequency of your diet, and please tick √ in the appropriate box

| **Food frequency** | **<1/month** | **1-3/month** | **1-3/week** | **4-6/week** | **Every day** |
| --- | --- | --- | --- | --- | --- |
| Staple food |  |  |  |  |  |
| Miscellaneous grains |  |  |  |  |  |
| Vegetables |  |  |  |  |  |
| Fruit |  |  |  |  |  |
| Fresh meat |  |  |  |  |  |
| Dairy products(milk) |  |  |  |  |  |
| Tofu and soy products |  |  |  |  |  |
| Eggs |  |  |  |  |  |
| Fried food |  |  |  |  |  |
| Spicy food |  |  |  |  |  |
| Smoked food |  |  |  |  |  |
| Fish and shrimp |  |  |  |  |  |

1. Which food you prefer to eat? (1) Less oil (2) General (3) greasy

2. Which your prefer to taste? (1) Light (2) Normal (3) Salty
