# Supplementary material for: A variant in KCNQ1 gene predicts metabolic syndrome among northern urban Han Chinese women
Source: BMC Med Genet. 2018 Aug 29;19:153. doi: 10.1186/s12881-018-0652-3 (PMC6114251; doi:10.1186/s12881-018-0652-3)
Supplement: Supplementary file 3 — Figure S1. the map (r2) of linkage disequilibrium of KCNQ1 gene SNPs rs231359, rs2237892, rs163182, rs2237895 and rs2237897. (DOCX 19 kb) [file 12881_2018_652_MOESM3_ESM.docx]

Additional file 3: Figure S1


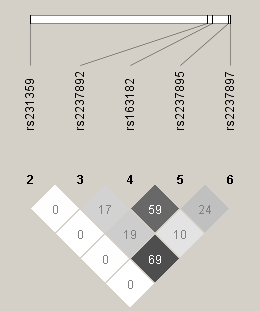


Figure S1 the map (*r^2^*) of linkage disequilibrium of KCNQ1 gene SNPs rs231359, rs2237892, rs163182, rs2237895 and rs2237897.
